# Raça/cor, gênero e classe nas relações sociais e afetivas de jovens negras: uma abordagem interseccional

**DOI:** 10.1590/0102-311XPT200424

**Published:** 2025-09-01

**Authors:** Gisele Maria Brito Lima, Simone Souza Monteiro, Regina Maria Barbosa, Andréa Fachel Leal, André Luiz Machado das Neves, Daniela Riva Knauth, Naiara Maria Santana Neves, Laio Magno

**Affiliations:** 1 Departamento de Ciências da Vida, Universidade do Estado da Bahia, Salvador, Brasil.; 2 Secretaria Municipal de Saúde, Prefeitura Municipal de Salvador, Salvador, Brasil.; 3 Instituto Oswaldo Cruz, Fundação Oswaldo Cruz, Rio de Janeiro, Brasil.; 4 Núcleo de Estudos de População, Universidade Estadual de Campinas, Campinas, Brasil.; 5 Faculdade de Medicina, Universidade Federal do Rio Grande do Sul, Porto Alegre, Brasil.; 6 Universidade do Estado do Amazonas, Manaus, Brasil.; 7 Universidade Estadual de Santa Cruz, Salvador, Brasil.; 8 Instituto Gonçalo Muniz, Fundação Oswaldo Cruz, Salvador, Brasil.; 9 Instituto de Saúde Coletiva, Universidade Federal da Bahia, Salvador, Brasil.

**Keywords:** Adolescente, Enquadramento Interseccional, Perspectiva de Gênero, Classe Social, População Negra, Adolescent, Intersectional Framework, Gender Perspective, Social Class, Black People, Adolescente, Marco Interseccional, Perspectiva de Género, Clase Social, Población Negra

## Abstract

A experiência de adolescer ocorre em contextos múltiplos mediada pelas posições que o indivíduo ocupa nas estruturas sociais. Objetivamos compreender o papel das interações entre marcadores sociais da diferença, como raça/cor, gênero e classe social, nas relações sociais e afetivas de jovens negras de um bairro popular em Salvador, Bahia, Brasil. Pesquisa qualitativa, recorte de um estudo multicêntrico realizado em cinco cidades do Brasil. Foram incluídos dados de 16 entrevistas e de um grupo focal realizados com adolescentes negras de 15 a 19 anos, no período de maio de 2021 a agosto de 2022. A análise do material empírico teve como referencial teórico a interseccionalidade. As vivências das adolescentes negras em espaços de sociabilidade de bairros populares, como os “paredões” e a escola, foram atravessadas pelo racismo, sexismo e discriminação de classe. Já as relações afetivas foram marcadas principalmente pela intersecção entre racismo e sexismo, que pretere e objetifica os corpos femininos negros. Nesse contexto de múltiplos atravessamentos, os espaços coletivos de resistência existentes no território despontam como equipamentos potentes na promoção de consciência racial e de gênero e na discussão de estratégias para a produção da equidade. Nesse contexto, evidencia-se que a intersecção entre os marcadores sociais de raça/cor, gênero e classe social atua na produção de múltiplas opressões contra adolescentes negras e periféricas nesses espaços e experiências afetivas.

## Abstract (en)

The experience of adolescence occurs in multiple contexts mediated by the positions these individuals occupy in social structures. We aim to understand the role of interactions between social markers of difference, such as race/color, gender, and social class, in the social and affective relationships of young black women in a working-class neighborhood in Salvador, Bahia State, Brazil. This qualitative research is based on a multicenter study in five Brazilian municipalities. Data from 16 interviews from May 2021 to August 2022 and a focus group conducted with black adolescents who were aged from 15 to 19 years were included. The analysis of the empirical material had intersectionality as a theoretical framework. The experiences of black adolescents in spaces of sociability in popular neighborhoods, such as *paredões* and the school, were crossed by racism, sexism, and class discrimination. Affective relationships, on the other hand, were mainly marked by the intersection between racism and sexism, which and objectifies black female bodies. In this context of multiple crossings, the collective spaces of resistance in the territory emerge as powerful equipment to promote racial and gender awareness and to discuss strategies to produce equity. This context evinces that the intersection between the social markers of race/color, gender, and social class acts in the production of multiple oppressions against black and peripheral adolescents in these spaces and affective experiences.

## Abstract (es)

La experiencia de la adolescencia se da en múltiples contextos mediados por las posiciones que el individuo ocupa en las estructuras sociales. Nuestro objetivo es comprender el papel de las interacciones entre los marcadores sociales de diferencias, como la raza/color, el género y la clase social, en las relaciones sociales y afectivas de jóvenes negras en un barrio de clase trabajadora en Salvador, estado de Bahía, Brasil. Esta investigación cualitativa se basa en un estudio multicéntrico realizado en cinco ciudades de Brasil. Se incluyeron los datos de 16 entrevistas y de un grupo focal realizado con adolescentes negras de 15 a 19 años entre mayo de 2021 y agosto de 2022. El análisis del material empírico tuvo como marco teórico la interseccionalidad. Las vivencias de las adolescentes negras en espacios de sociabilidad en barrios populares, como los “paredões” [fiestas callejeras con música a alto volumen] y la escuela, estuvieron atravesadas por el racismo, el sexismo y la discriminación de clase. Las relaciones afectivas, por otro lado, estuvieron marcadas principalmente por la intersección entre el racismo y el sexismo, que desprecia y cosifica los cuerpos femeninos negros. En este contexto en que multiples factores interactúan, los espacios colectivos de resistencia existentes en el territorio emergen como poderosos equipamientos en la promoción de la conciencia racial y de género y en la discusión de estrategias de construcción de la equidad. En este contexto, es evidente que la intersección entre los marcadores sociales de raza/color, género y clase social actúa en la producción de múltiples opresiones contra las adolescentes negras y periféricas en estos espacios y experiencias afectivas.

## Introdução

A juventude, fase de desenvolvimento biopsicossocial entre 15 e 24 anos ^1^, abrange múltiplas experiências mediadas por classe, raça/cor, gênero e outras posições nas estruturas sociais ^2^ ^,^ ^3^ ^,^ ^4^ ^,^ ^5^. No Brasil, adolescentes negras enfrentam maior vulnerabilidade que brancas. O Fundo das Nações Unidas para a Infância (UNICEF), com base em dados da *Pesquisa Nacional por Amostra de Domicílios Contínua* (PNAD Contínua) de 2021, aponta que mais de 50% das crianças e jovens negros brasileiros vivem em privação de renda, enquanto essa situação atinge menos de 30% das crianças brancas e amarelas ^6^. Essa privação reflete processos históricos e sociais de subjugação da população negra, resultando em posições desfavorecidas na estrutura social e moradias em áreas com pouca presença do Estado ^7^ ^,^ ^8^.

Além disso, jovens negras são afetadas de forma desproporcional pela violência física e sexual em comparação às brancas. Dados do Sistema de Informações sobre Mortalidade (SIM) de 2019 indicam que 68% dos óbitos entre jovens negras de 15 a 19 anos ocorreram por agressão, enquanto entre jovens brancas da mesma faixa etária essa taxa foi de 52% ^9^. Entre 2015 e 2021, estudo de série temporal revelou que jovens negras representaram 60,3% dos casos de violência sexual notificados nesse período ^10^. Esses dados de violência desproporcional trazem à tona um fenômeno complexo, abordado sob a perspectiva teórico-metodológica da interseccionalidade, oriunda dos estudos feministas de raça e gênero no Brasil e nos Estados Unidos entre as décadas de 1980 e 1990, para compreender as múltiplas formas de marginalização enfrentadas por mulheres negras ^11^.

No Brasil, o Movimento Negro liderado por intelectuais feministas negras, como Lélia Gonzalez e Sueli Carneiro, criticou o feminismo hegemônico e evidenciou a importância de discutir a mulher para além do gênero, compreendendo o cruzamento das avenidas identitárias (e.g., raça/cor, gênero e classe social) e seus impactos sobre os corpos e direitos sociais e políticos de mulheres negras ^12^ ^,^ ^13^. Nesse mesmo período, os Estados Unidos vivenciavam cenário semelhante, buscando aprofundar a compreensão das múltiplas experiências de marginalização vivenciadas por mulheres negras, a partir das variadas identidades que se entrecruzam (e.g., negras, mulheres, pobres etc.) ^11^.

A interseccionalidade aprofunda a compreensão das iniquidades em saúde, ao possibilitar a análise de marcadores sociais da diferença - como raça/cor, gênero e classe social - que se articulam para produzir sistemas de privilégio e opressão ^14^. Essa perspectiva permite entender a complexidade das relações de poder, questionando como esses marcadores atuam nos âmbitos individual, institucional e estrutural, com o objetivo de promover mudanças sociais e combater opressões ^15^ ^,^ ^16^.

Nesse sentido, estudos documentam o papel dos marcadores sociais da diferença, como raça/cor, gênero e classe social, na saúde de jovens negras. Pesquisa qualitativa e quantitativa com 816 jovens moradoras de dez favelas do Rio de Janeiro, em 2010, revelou que 74% delas se identificavam como negras, e que a discriminação racial cotidiana contribuía para uma autoimagem negativa, agravando a vulnerabilidade às infecções sexualmente transmissíveis (ISTs) devido ao sexismo, pobreza e barreiras programáticas ^17^. Esses marcadores influenciam na produção de desigualdades e exclusão social ^15^, impactando diretamente a saúde.

Embora outras pesquisas tenham explorado as vivências de jovens negras em contextos de alta vulnerabilidade social, ainda há poucos estudos que utilizem perspectiva interseccional, considerando simultaneamente os marcadores de raça/cor, gênero e classe social. Assim, esta investigação busca compreender o papel das interações entre esses marcadores nas relações sociais e afetivas de jovens negras em um bairro popular de Salvador, Bahia, Brasil.

## Métodos

Este subestudo qualitativo faz parte de uma pesquisa maior que analisa contextos de exposição ao HIV/ISTs entre adolescentes de 15 a 19 anos em bairros populares de Porto Alegre (Rio Grande do Sul), São Paulo, Salvador (Bahia), Manaus (Amazonas) e Rio de Janeiro. Os critérios de inclusão foram idade entre 15 e 19 anos e residência no bairro pesquisado, com autodeclaração como negra (preta ou parda, conforme o Instituto Brasileiro de Geografia e Estatística − IBGE).

Foi desenvolvido em um bairro localizado no Distrito Sanitário Cabula-Beiru, no centro geográfico de Salvador. Pertence a um território quilombola e tem sua história marcada por lideranças religiosas de terreiros. Alguns equipamentos sociais presentes no território são: escolas, creches, igrejas, terreiros e unidades de saúde ^18^.

As adolescentes foram recrutadas em uma unidade de saúde da família (USF) e em locais de convivência, como escolas e em um grupo organizado pela USF focado em empoderamento feminino e saúde sexual. O estudo ocorreu em um bairro quilombola em Salvador, caracterizado por alta vulnerabilidade social, uma população majoritariamente negra e feminina ^19^, com problemas de acesso a serviços básicos e altos índices de violência devido a racismo institucional e disputas de facções ^18^.

Entre maio e dezembro de 2021, foram realizadas 16 entrevistas semiestruturadas até atingir a saturação dos dados ^20^, conduzidas por três pesquisadoras, todas mulheres cis, sendo duas enfermeiras da USF do bairro pesquisado, uma autodeclarada parda e outra branca, e uma antropóloga parda. Foram abordados temas como dados sociodemográficos, comportamento sexual, discriminação e ISTs, gravados e transcritos para análise.

Para aprofundar aspectos sobre questões raciais e relações afetivas, realizou-se um grupo focal com 12 jovens negras, em agosto de 2022, na sala de uma escola do bairro. A discussão incluiu raça/cor, saúde sexual e práticas preventivas. Destas, duas também haviam participado da etapa das entrevistas. Para facilitar a interação e observação das participantes, as cadeiras foram dispostas em formato de semicírculo; as moderadoras se posicionaram à frente e as observadoras no fundo da sala, registrando gestos, expressões e interações. O roteiro utilizado para a condução do grupo contemplou perguntas disparadoras sobre: raça/cor, saúde sexual e reprodutiva e práticas de prevenção. O grupo focal teve a duração média de uma hora e quarenta minutos. Para a captação do áudio, foram utilizados um gravador e um celular. A transcrição foi realizada posteriormente por uma assistente de pesquisa treinada.

A análise de dados seguiu a técnica de análise de conteúdo temática, com três fases: sistematização inicial, exploração e tratamento dos resultados ^21^ ^,^ ^22^. As categorias analíticas foram divididas em éticas (gênero, classe e raça/cor) e êmicas (racismo, discriminação e violência no território), discutidas sob a perspectiva interseccional. Essas categorias foram discutidas em articulação com o referencial teórico, para dar sentido à interpretação e responder as questões levantadas na pesquisa.

O estudo seguiu a *Resolução nº 466/2012* e foi aprovado pelo Comitê de Ética da Universidade do Estado da Bahia (parecer nº 4.540.024). Aquelas com idade menor de 18 anos leram e assinaram o Termo de Assentimento; aquelas com idade maior de 18 anos leram e assinaram o Termo de Consentimento Livre e Esclarecido (TCLE). Nesta pesquisa, foi solicitada a dispensa do TCLE dos pais ou responsáveis.

## Resultados

### Característica geral das participantes

Participaram do estudo 26 meninas cisgêneras, 16 na etapa de entrevistas e 12 na etapa do grupo focal (duas participaram de ambas etapas). Não houve coleta de dados sociodemográficos entre as meninas dos grupo focal. Entre aquelas que participaram das entrevistas, como indica a Tabela 1, todas se declararam cisgênero, com idade entre 15 e 16 anos. Houve predomínio da autodeclaração preta (13) seguida de parda (3). Quinze se declararam heterossexuais e apenas uma bissexual. Todas solteiras, mas oito relataram algum tipo de relacionamento: cinco referiram estar namorando e três usaram os termos “conhecendo um parceiro”, “quase namorando” e “ficando”.

**Tabela 1:** Características das jovens negras que participaram da etapa das entrevistas. Salvador, Bahia, Brasil, 2022.

Variáveis	n	%
Idade (anos)		
15	4	25,0
16	5	31,3
17	4	25,0
18	2	12,5
19	1	6,2
Raça/cor		
Preta	13	81,3
Parda	3	18,7
Orientação sexual		
Heterossexual	15	93,8
Bissexual	1	6,2
Tem namorado(a)		
Sim	8	50,0
Não	8	50,0
Ocupação		
Só estuda	13	81,3
Só trabalha	1	6,2
Trabalha e estuda	1	6,2
Nem estuda e nem trabalha	1	6,2
Escolaridade		
Ensino Fundamental incompleto	7	43,8
Ensino Médio incompleto	8	50,0
Ensino Médio completo	1	6,2
Distorção idade-série (anos)		
Sem atraso	2	12,5
Atraso de 1	1	6,2
Atraso de 2	6	37,5
Atraso de 3	4	25,0
Atraso de 4 ou mais	3	18,8
Escolaridade materna		
Ensino Fundamental incompleto	5	31,3
Ensino Médio incompleto	4	25,0
Ensino Médio completo	4	25,0
Não sabe informar	3	18,7
Com quem mora		
Com a mãe/Com a mãe e irmão(s)	6	37,5
Com a mãe, o pai e irmão(s)	2	12,5
Com a mãe e filho	1	6,2
Com a mãe e padrasto/com a mãe, padrasto e irmão(s)	3	19,0
Com a mãe e avós	1	6,2
Com pai, madrasta e irmão(s)	1	6,2
Com avós	1	6,2
Sozinha	1	6,2

Fonte: elaboração própria.

A Tabela 1 apresenta as características das jovens negras participantes das entrevistas. No momento da entrevista, a maioria (13) das meninas referiu apenas estudar. Em relação à distorção idade-série, a maioria (14) apresentou atraso escolar. Já no quesito escolaridade materna, a maioria (9) declarou que a mãe não havia concluído o Ensino Fundamental e Médio. Foi frequente a presença materna em contraposição à ausência paterna: 13 meninas citaram a mãe no núcleo familiar e somente três relataram a presença do pai.

### “Você é favelada, você é ‘foveira’”: relações entre racismo e discriminação de classe social no território

As narrativas das meninas negras destacaram a interseccionalidade entre raça/cor e espaço geográfico. Notamos que o preconceito não era apenas baseado na raça, mas era amplificado pelo local de moradia. Viver em favela ou comunidade de alta vulnerabilidade social pode intensificar a discriminação racial, sugerindo que a estigmatização do espaço contribui para reforçar estereótipos raciais.

“*Raça vai também do local que a pessoa mora* (...) *eu acho que o preconceito vem mais, é mais forte quando a pessoa vem da favela, quando a pessoa mora na comunidade, que o povo julga assim: ah, porque naquela favela tem isso e isso*” (grupo focal).

“*Determina a favela toda por uma pessoa* (...) *ah, ali fulano de tal, aquele bairro não presta*” (grupo focal).

Identificamos que os termos “favelada” e “foveira” foram utilizados pelas meninas negras nas narrativas para se referirem aos moradores do bairro. Ao recorrerem a esses termos, observamos um processo de significação na identidade delas e no espaço que elas habitam que pode ter sido produzido em suas experiências dentro e fora da comunidade.

Além disso, foi relatada a generalização de rótulos estigmatizantes aos habitantes desses espaços, como, por exemplo, a prática de ações delituosas (roubos, tráfico etc.).

“...*quando uma pessoa negra entra no mercado já chama logo o segurança, achando que vai assaltar*” (grupo focal).

“...*você tá dentro do shopping, você é negro e o segurança negro começar a seguir você*” (grupo focal).

As narrativas acima apontam para a violência simbólica forjada pela segregação social, de olhares seletivos e vigilantes, especialmente quando há mobilidade em locais privados, fora do bairro onde moram. Isso reforça a intersecção entre racismo e discriminação por classe social, que relaciona pessoas negras com o potencial perigo de prática delituosa. A experiência da violência é relatada com mais pesar, quando reproduzida por pessoas da mesma cor e do mesmo território em posição de poder.

### “A gente corre quando a polícia chega, atirando pra cima e jogando bomba”: experiências de violência e discriminação no território

O território, rotulado como “favelado”, é relatado como locus de exposição à violência. Estas são produzidas pelo crime organizado, principalmente guerras entre facções distintas, ações policiais e confrontos entre esses dois grupos, o que provoca uma sensação de insegurança e limitação da livre circulação das adolescentes entre os bairros da região, especialmente pelo medo de represálias.

“*Só é ruim por causa do tráfico de drogas, por isso… fora isso, é bom*” (Crisântemo, 17 anos).

“*Eu não me sinto segura, porém eu vou* [passeio e festas em outros bairros] (...) *Porque, a maioria das vezes né…, tem lugar que tem ‘rixa’ ou algo do tipo assim, que a gente não pode ir, né?*” (Margarida, 17 anos).

Contudo, notamos uma dualidade na percepção do bairro: enquanto a presença do tráfico de drogas é vista como um aspecto negativo significativo, outros elementos são valorizados. Isso pode indicar uma resiliência comunitária e uma valorização dos aspectos sociais e culturais do bairro, apesar dos desafios impostos pela criminalidade.

Ainda no tocante à dimensão violência, as narrativas desvelam que os conflitos entre a polícia e as facções são relatados com sentimento de medo e insegurança. Não foram raros os depoimentos de tiroteios, balas cravejando as paredes das casas, arma sendo apontada para cabeça e outras cenas de violência, especialmente nos territórios de maior vulnerabilidade social do bairro.

“...*no meu quarto tem muitos furos de bala, então* (...) *toda vez que rola tiro lá* [próximo de casa] *eu sempre me abaixo* (...) *da última vez que eu fui botar o olho na janela, teve um policial* (...) *com o raio da arma dele em direção à minha testa* (...). *Teve uma vez que os traficantes tava lá no portão e pulou, bateu na minha porta e caiu assim de costas. Então, eu fico com medo, porque, no bairro onde eu moro, eu não me sinto segura*” (Rosa, 16 anos).

A narrativa acima ainda aponta duas dimensões na experiência das interlocutoras. A invasão do ambiente doméstico, tradicionalmente compreendido como um espaço de segurança, violado pela violência externa, amplia a sensação de insegurança e afeta a saúde mental e emocional das jovens negras. Outra dimensão é a dupla ameaça representada pela violência policial e do tráfico de drogas, forças opressoras que interagem para criar um ambiente hostil e inseguro.

Compreendemos que a insegurança e a violência são presentes nas vidas dessas jovens, tanto dentro de suas casas quanto em espaços públicos. A violência policial e a presença do tráfico de drogas não só invadem o espaço doméstico, como também moldam a mobilidade e as oportunidades de sociabilidade.

### “Só o paredão mesmo”: sociabilidade juvenil e a violência policial na diversão

Em relação aos espaços de sociabilidade juvenil, o relato é de escassez de opções. O “paredão” foi frequentemente reconhecido como a única ou principal alternativa de festas para sociabilização no território, tendo como fatores potencializadores a proximidade local e a insuficiência de recursos dos jovens para acessar outros lugares. A sonorização desses eventos, descritos como festas de rua, é feita por aparelhos de som potentes verticalizados e dispostos no fundo de carros. Nessas festas, os jovens dançam, usam álcool e outras substâncias psicoativas, relacionam-se afetiva e sexualmente e, algumas vezes, há a presença de representantes locais de venda de drogas.

As festas do tipo paredão são realizadas principalmente em dois locais: em uma quadra próxima à escola, este reconhecido como menos “perigoso”; e em outro espaço, local X, perto do final de linha do bairro. Esse local é conhecido por ser mais vulnerável socialmente, ter maior presença do crime organizado e ser de “maior periculosidade”, devido às abordagens policiais, quase sempre inibitórias e arbitrárias, contra seus frequentadores.

Durante a pandemia, as adolescentes indicaram que as incursões policiais violentas nas festas de paredão foram intensificadas, principalmente no espaço de maior vulnerabilidade. Houve o relato de uso de bombas de gás e disparos de armas de fogo, que provocaram percepções de medo e desespero, comprometendo a segurança e a diversão dos jovens.

“*Porque, paredão, você tá se acabando, dançando, e quando você vai ver, os policial* [chegam] *tudo atirando, jogando bomba, todo mundo correndo*” (Girassol, 16 anos).

As adolescentes descreveram a repressão policial em eventos culturais e de lazer no bairro. As festas de paredão, espaços importantes de sociabilidade e expressão cultural entre adolescentes e jovens, são frequentemente interrompidas de maneira violenta pela polícia. A descrição de tiros e bombas transforma um ambiente de diversão em um cenário de pânico, refletindo a criminalização da cultura e do lazer de pessoas negras.

### “A mulher é mais dominada pelo cabelo e aparência”: entrelaçamentos entre racismo e sexismo, e experiências de violência sexual

Esse processo fica evidente quando características relacionadas à aparência física são acionadas no discurso sobre estética e beleza. O cabelo “duro” e as “tranças” são rotulados como estigma das meninas negras e, portanto, como elemento de desvalorização em relação à estética hegemônica branca, que exclui traços fenotípicos negros e interfere diretamente nas relações de sociabilidade.

“*Ah, a menina passou me chamou de preta, falou que eu tinha cabelo duro* (...) *eu fiquei muito triste, fiquei uns dias deprimida, não queria comer nem sair do quarto*” (Lírio, 16 anos).

“*As pessoas acha que negra tem que ter o cabelo duro*” (grupo focal).

A fala acima reflete estereótipos raciais específicos sobre a aparência das pessoas negras. A expectativa de que “negra tem que ter o cabelo duro” mostra como as experiências narradas trazem os efeitos das normas sociais racistas que moldam a percepção das características físicas associadas às mulheres negras. De maneira geral, as falas do grupo focal refletem como se reproduz a discriminação racial, o modo como se normalizam e perpetuam estereótipos que desvalorizam a diversidade de aparências das meninas negras interlocutoras.

Identificamos, ainda no grupo focal, que as primeiras experiências com o racismo se deram ainda na infância, por meio da percepção de exclusão e do não pertencimento aos grupos no ambiente escolar e aos locais de “barões”. “Barão” é um termo geralmente atribuído às pessoas de alto status socioeconômico, reconhecidas frequentemente como brancas, o que sugere uma estreita relação entre raça e classe social.

“*Na escola mesmo* (...) *quando era pequena, a gente sempre era excluída por causa que ficava aquele grupinho* (...) *ah, eu não quero brincar com você porque você é assim de tal forma* (...) *É... você é diferente de mim, então eu não quero brincar com você*” (grupo focal).

“*...era aniversário de barão, né? Que vocês sabem que barão geralmente tem filho branquinho, dinheiro* [risos]. *Aí eu cheguei lá e eu tava toda bonitinha* (...). *Aí o povo olhava assim... Eu via as mulher me olhando assim por eu ser negra*” (grupo focal).

O racismo é abordado como parte da experiência cotidiana em espaços públicos em geral, porém é mais prevalente e naturalizado no ambiente escolar, ainda que a escola pertença ao território e seja ocupada predominantemente por pessoas negras. As ofensas e os olhares de julgamento relacionados à cor da pele e ao cabelo “duro” indicam práticas discriminatórias percebidas pelas jovens. A repetição contínua da discriminação e violência racial interseccionada com aquelas relacionadas à classe social atuam sinergicamente para ampliação do sofrimento mental. Foram vários os relatos de sentimentos de tristeza, perplexidade e raiva, bem como isolamento e dificuldade com a autoestima.

“...*sobre o cabelo, já falou* [pessoas desconhecidas] *que era duro, sabe? Em lugares assim, público* (...). *Eu fiquei chocada, né? Muito triste pela pessoa ter falado aquilo e também não tive reação no momento pra falar*” (Margarida, 17 anos).

“...*eu não saio muito de casa, porque tem alguns meninos aqui que fica de preconceito comigo* (...) *teve um menino que me chamou de macaca* (...) *e falou que eu sou feia*” (Rosa, 16 anos).

“*Eu tinha o cabelo cacheado, crespo. Eu alisei* (...) *por vários comentários ruins que teve* (...) *‘ah, cabelo duro’, ou pelo fato de muita gente dizer que dá muito trabalho* (...). *Eu preferi alisar até pra eu me sentir melhor* (...) *me sentir mais arrumada e mais dentro do padrão*” (grupo focal).

Nesse contexto de racismo, a trajetória das jovens negras também foi relatada como atravessada pela exposição à violência sexual. A violação desses corpos ocorre em condições desiguais de poder, por exemplo, situações em que o agressor é homem e dotado de uma autoridade familiar ou simbólica. O abuso sexual foi frequente, sobretudo na infância, e teve, como principais perpetradores, familiares e conhecidos. As dores das violências foram mantidas em silêncio por pressuporem que os familiares não confiariam em seus relatos, uma vez que o agressor pertencia ao seio familiar e aparentava ser isento de suspeitas. Apesar de a maioria das violências ter ocorrido na infância, a convivência com o abusador e a memória do trauma mantêm-se presentes.

“*Eu tinha sete anos* (...) *meu padastro* [sic] *também me abusou* (...). *Fingia que o controle tava no chão e passava a mão nas minhas partes íntima, pegava no meu peito* (...) *o cara depois de tudo chegou e falou pra ela [mãe] que ele já me abusou e minha mãe ficou ‘porque você não me falou?’, e eu peguei e falei ‘porque eu era pequena e ele disse que ia matar eu e a senhora’*” (Rosa, 16 anos).

“*Acho que tinha uns 12, 13 anos por aí e tals, que foi pessoa da minha família e tals, tava dormindo* (...) *despertei, eu percebi que tava me tocando e tal, e como eu não sabia o que fazer, eu fiquei sem reação e não fiz nada*” (Jasmim, 15 anos).

“*Não, nunca conversei com ninguém sobre* (...) *porque também acredito que não acreditaria, por ser com aquela pessoa, a situação* (...) *acho que a pessoa* [avó, pais] *nem ia acreditar* (...)” (Margarida, 17 anos).

### “Ah, você é mais negra que ele”: relações afetivas, sexismo e racismo

As adolescentes associaram a cor de pele branca e o cabelo liso, isto é, o modelo estético branco hegemônico, ao maior despertar do desejo sexual masculino. Isso sugere que as relações afetivas também são influenciadas pelo racismo, sendo nítida a percepção das meninas sobre essa preferência dos meninos em detrimento das características fenotípicas negras. A ideia de “preferência” aparece sempre nas falas como exclusividade dos meninos. As meninas negras, geralmente, percebiam-se como menos “desejadas” ou menos “preferidas” em relação às meninas brancas. Essa posição de objeto do desejo dos meninos devido a aparência fenotípica sugere sexismo.

“*Eles sempre vão preferir você, pela sua aparência* (...) *por você ser branca, mais bonita, ter o cabelo liso*” (grupo focal).

No contexto de relacionamentos interraciais, percebemos que as meninas negras eram confrontadas pelo racismo existente na sociedade, seja de forma explícita ou velada. Esse fenômeno também parece interferir diretamente no processo de escolha das parcerias afetivas por conta do medo do julgamento relacionado à discriminação racial.

“*Tipo assim, eu sempre me relacionei mais com pessoas da minha cor do que com pessoas de outra cor, porque, geralmente, assim… as pessoas olham assim*” (grupo focal).

“*Mas sempre tem alguém que vai dizer:* ‘(...) *ah, você é mais negra que ele, ele é mais branco que você’*” (grupo focal).

### “Tá mais aberto conversar sobre raça (...) a gente aprende e passa pra nossos filhos”: o diálogo como estratégia de enfrentamento ao racismo e sexismo

As meninas trouxeram que as discussões sobre raça e racismo na sociedade tinham reverberado de forma positiva na afirmação da identidade negra na comunidade. Isso sugere a existência de um processo de mudança em curso, que tem elevado a consciência de autopertencimento étnico-racial na comunidade negra e reforça uma postura de enfrentamento ao racismo.

“*Na geração de hoje em dia, as meninas* (...) *se considera como preta mesmo. Antigamente, você falava: ‘ah, não, eu não sou preta, sou morena’. Hoje em dia, o pessoal fala mais a palavra preto: “eu sou preta*” (grupo focal).

“*Muitas dizem assim: ‘ah, não, que eu também me considero preta’. Tipo assim, pra poder se autodenominar, porque, hoje em dia, esse assunto tá muito forte*” (grupo focal).

“*Hoje, nessa geração que o povo conversa… antigamente não podia conversar sobre raça na sala de aula porque você tinha algum problema* (...)” (grupo focal).

A constituição de espaços de diálogo em instituições sociais, como escolas e projetos sociais, e a repercussão na mídia de pautas raciais representam estratégias de enfrentamento de posturas e atitudes racistas para ampliar as vozes no combate à discriminação. Para as meninas, a possibilidade de debater abertamente sobre raça na sala de aula é entendida como um ponto positivo, pois a reflexão traz aprendizados que contribuirão para repercussões positivas intergeracionais. O grupo da USF, por exemplo, uma estratégia de promoção da saúde para o empoderamento feminino, com foco nas discussões de raça e gênero, é reconhecido por algumas adolescentes como um forte equipamento social na produção de agência e consciência coletiva para a adoção de posturas de enfrentamento ao racismo e sexismo.

“*Aonde eu tirei mais o conhecimento e tudo mais, foi no* [grupo da USF] (...). *Que aí a gente conversava de tudo, falava sobre tudo* (...)” (Tulipa, 17 anos).

## Discussão

Observamos que as interações entre racismo, sexismo e discriminação de classe social na experiência de meninas negras produzem processos de vulnerabilização. Nossa pesquisa também apontou que o bairro foi percebido como um espaço estigmatizado e marcado pela violência policial e do tráfico. Essa violência revela a falha do Estado em garantir direitos básicos, como segurança, proteção social e cidadania. Estudo qualitativo realizado em Salvador, Fortaleza (Ceará) e Recife (Pernambuco), em 2020, em bairros periféricos, com jovens negros, de ambos os sexos, de 15 a 29 anos, documentou que, nas três cidades, todos(as) os(as) jovens autodeclarados(as) negros(as) referiram já terem sido abordados(as) pela polícia ao menos uma vez na vida, e a maioria relatou vivenciar essa experiência com muita frequência ^23^.

Grande parte das abordagens policiais arbitrárias em Salvador estava diretamente relacionada a marcadores raciais e de pertencimento territorial, o que explicita o racismo e a discriminação relacionados à classe social, praticados pela instituição armada estatal. Em Recife, a raça/cor e a aparência, especificamente a vestimenta, foram os principais fatores. Em Fortaleza, foram enfatizados o marcador de classe social e o pertencimento territorial ^23^.

A produção de violência nos territórios negros, que associa espaço de morada com a raça/cor, é denominada de “necropolítica espacial” por Alves ^24^ ^,^ ^25^. Nessa perspectiva, a raça/cor define a lógica espacial e o direito à vida ou morte nos episódios de violência ou omissão praticados pelo Estado. Gonzales ^26^ ressalta que a repressão policial nesses territórios visa impedir a unidade do grupo dominado e garantir sua divisão interna.

A violência também invade espaços de socialização frequentados pelas jovens negras. Outro trabalho com jovens mostrou fenômeno semelhante na periferia da cidade de São Paulo, em que participantes referiram que o lazer no baile funk era continuamente criminalizado e violentado pela polícia ^5^.

Apesar das experiências de violência, neste estudo, as jovens informaram haver poucas alternativas de diversão além do “paredão”. Ademais, a circulação para outros espaços da cidade era limitada pela renda insuficiente. Situação similar foi mostrada num estudo interseccional com meninas e meninos de 15 a 17 anos de uma escola pública da periferia de São Paulo, em 2014, em que jovens referiram não ter acesso à cultura em outros locais da cidade ^27^. Assim, experiências de socialização são moldadas pelo território. Demais investigações mostram que jovens negros de bairros percebidos como “violentos” são afetados por rótulos como “perigoso”, “criminoso” etc. ^28^ ^,^ ^29^, “favelado” ^5^, “preto” e “pobre” ^27^, evidenciando o racismo e a discriminação de classe.

Dessa forma, a intersecção entre classe social e raça/cor contribui para o apagamento da identidade individual das jovens, substituída por uma identidade coletiva estigmatizada atribuída ao território periférico. Essa violência simbólica é constantemente reproduzida com o objetivo de reiterar o lugar social reservado a elas.

As meninas do nosso estudo percebiam a escola como um ambiente hostil. Embora seja considerada lócus de socialização para a promoção da emancipação e diversidade, percebemos que, em muitas realidades, ela adota, em suas práticas pedagógicas, o modelo hegemônico eurocêntrico. Essa política educacional epistemicida invisibiliza as contribuições culturais e sociais fora do modelo europeu, dificultando a identificação das tensões raciais e de gênero presentes no seu ambiente ^30^. Dessa forma, surge um ambiente propício para a produção e (re)produção do racismo desde a mais tenra idade entre as meninas negras.

Pesquisa qualitativa com cinco jovens negras de 18 a 23 anos, em Contagem e Belo Horizonte, corrobora o resultado ao demonstrar que o racismo esteve presente no espaço escolar das participantes, com maior evidência entre as meninas e no ensino fundamental, o que revela uma intersecção de racismo e sexismo ^31^. Outro estudo qualitativo, realizado em São Paulo, com mulheres negras, em 2021, mostrou que as experiências de racismo no ambiente escolar foram frequentes durante a infância, especialmente por conta da aparência do cabelo ou da cor de pele ^32^.

Assim, a racialização das meninas negras é imposta desde suas primeiras vivências em espaços de sociabilidade. Essas experiências ocorrem pela introjeção de sentimentos de inferioridade em relação ao padrão estético hegemônico branco, resultando em rejeição da identidade racial e estratégias de apagamento dos traços étnico-raciais, especialmente o alisamento do cabelo crespo, reconhecido como forte símbolo identitário ^31^ ^,^ ^32^ ^,^ ^33^ ^,^ ^34^. A insatisfação com a autoimagem e textura do cabelo representa mais que um comportamento habitual da juventude, pois é agravada pelo aspecto racial ^33^ ^,^ ^34^. O racismo provoca sentimentos de auto-ódio, oprime o direito à imagem ou representação positiva, e reduz possibilidades no campo afetivo-sexual ^35^.

Em nossa pesquisa, observamos que o preterimento afetivo, devido às características fenotípicas negras, está presente desde o início da adolescência. Gonzalez ^12^, Carneiro ^35^ e Kilomba ^36^ abordam os atravessamentos do racismo e patriarcado que, desde o período colonial, produziram e perpetuaram estereótipos que objetificam a mulher negra e provocaram a naturalização da sua subvalorização e das desigualdades nas relações de gênero.

Resultado semelhante é relatado por Moutinho et al. ^37^, em estudo sobre mulheres negras e experiências afetivas em São Paulo. Estudo de Reis & Ribeiro ^38^, com jovens e adultos das regiões “centrais” e “periféricas” de Belém (Pará), também demonstrou que a raça/cor branca permanece como ideal ou preferencial para relações afetivas, principalmente para relacionamentos sérios.

Assim, a associação dos marcadores de raça e gênero produz maior exclusão das mulheres negras nas vivências afetivas ^39^. Como escolhas afetivas são reguladas por práticas culturais e influenciadas pelo racismo estrutural, representações sociais dessas mulheres são de corpos hipersexualizados, racializados e não dignos de afeto, reservando-lhes a solidão ^35^ ^,^ ^39^ ^,^ ^40^ ^,^ ^41^. Ademais, nosso estudo também demonstrou que o sexismo associa-se ao racismo na esfera afetiva: ao homem é concedido o poder de escolher, enquanto à mulher é relegado o lugar de objeto sexual e subordinação aos desejos masculinos.

Nesse cenário de objetificação da mulher, somam-se as frequentes experiências de violência sexual relatadas pelas jovens. Entre os anos de 2015 e 2021, no Brasil, foi levantado que a maioria (76,9%) dos casos de violência sexual na infância teve, como vítima, meninas negras na faixa etária de 5 a 9 anos, enquanto que, na adolescência, foi mais comum entre 10 e 14 anos, sendo 92,7% meninas e, destas, 60,3% eram negras ^10^. Dessa forma, observamos que a violência sexual é potencializada pela intersecção de raça/cor, gênero e classe social, posicionando meninas negras e periféricas na base da escala do poder e ampliando sua vulnerabilidade ^42^. Isso ocorre, porque, historicamente, essas meninas têm seus direitos invisibilizados e são vítimas da “adultização” desde a infância, expondo-as às mesmas opressões vivenciadas por mulheres adultas negras ^43^.

Nesse contexto de múltiplas violências, os espaços coletivos de resistência presentes no território, como os debates nas escolas e no grupo da USF, foram apontados como pontos positivos na promoção de consciência racial e de gênero, fortalecimento do reconhecimento étnico/racial e discussão de estratégias para a produção da equidade. Resultado semelhante foi encontrado na pesquisa qualitativa sobre estratégias de enfrentamento ao racismo e à opressão, realizada em 2018, com cinco mulheres, com idades entre 30 e 53 anos, autodeclaradas negras e militantes de movimentos sociais, da região centro-oeste de Minas Gerais. O estudo apontou a importância dos espaços coletivos de resistência, para dar visibilidade às lutas, bem como o reconhecimento e a valorização da ancestralidade e do investimento no conhecimento para qualificação do debate e enfrentamento das desigualdades ^44^.

## Considerações finais

O estudo evidencia que meninas negras nas periferias enfrentam trajetória marcada por racismo, violência, exclusão social e afetiva, e violações de direitos, resultando em impactos negativos na saúde mental e relações sociais. Instituições como o Estado, a escola e a família frequentemente reforçam essas opressões, falhando em garantir um ambiente saudável e de apoio ao desenvolvimento dessas jovens. O reconhecimento das opressões baseadas em raça, gênero e classe social pode fomentar estratégias de resistência e demanda por políticas públicas intersetoriais de equidade, além de fortalecer espaços de debate e empoderamento, como escolas, serviços de saúde e centros comunitários.
